# Chitosan-GSNO nanoparticles: a positive modulator of drought stress tolerance in soybean

**DOI:** 10.1186/s12870-023-04640-x

**Published:** 2023-12-11

**Authors:** Nusrat Jahan Methela, Anjali Pande, Mohammad Shafiqul Islam, Waqas Rahim, Adil Hussain, Da-Sol Lee, Bong-Gyu Mun, Nirmal Prashanth Maria Joseph Raj, Sang-Jae Kim, Yoonha Kim, Byung-Wook Yun

**Affiliations:** 1 Department of Applied Biosciences, College of Agriculture and Life Sciences, Kyungpook National University, Daegu, 41566 South Korea; 2 Department of Agriculture, Noakhali Science and Technology University, Noakhali, 3814 Bangladesh; 3 Department of Agriculture, Abdul Wali Khan University Mardan, Khyber Pakhtunkhwa, Mardan, 23200 Pakistan; 4 Nanomaterials and Systems Lab, Mechatronics Engineering, Faculty of Applied Energy System, Jeju National University, Jeju, 63243 South Korea; 5 Energy Harvesting Research Group, School of Physics & Astronomy, SUPA, University of St Andrews, St. Andrews, Fife, KY16 9SS UK

**Keywords:** *Glycine max*, Chitosan, Nanoparticles, GSNO, Nitric oxide

## Abstract

**Background:**

Chitosan biopolymer is an emerging non-toxic and biodegradable plant elicitor or bio-stimulant. Chitosan nanoparticles (CSNPs) have been used for the enhancement of plant growth and development. On the other hand, NO is an important signaling molecule that regulates several aspects of plant physiology under normal and stress conditions. Here we report the synthesis, characterization, and use of chitosan-GSNO nanoparticles for improving drought stress tolerance in soybean.

**Results:**

The CSGSNONPs released NO gas for a significantly longer period and at a much lower rate as compared to free GSNO indicating that incorporation of GSNO in CSNPs can protect the NO-donor from rapid decomposition and ensure optimal NO release. CS-GSNONPs improved drought tolerance in soybean plants reflected by a significant increase in plant height, biomass, root length, root volume, root surface area, number of root tips, forks, and nodules. Further analyses indicated significantly lower electrolyte leakage, higher proline content, higher catalase, and ascorbate peroxidase activity, and reduction in MDA and H_2_O_2_ contents after treatment with 50 μM CS-GSNONPs under drought stress conditions. Quantitative real-time PCR analysis indicated that CS-GSNONPs protected against drought-induced stress by regulating the expression of drought stress-related marker genes such as *GmDREB1a*, *GmP5CS*, *GmDEFENSIN*, and NO-related genes *GmGSNOR1* and *GmNOX1*.

**Conclusions:**

This study highlights the potential of nano-technology-based delivery systems for nitric oxide donors to improve plant growth, and development and protect against stresses.

**Supplementary Information:**

The online version contains supplementary material available at 10.1186/s12870-023-04640-x.

## Background

Drought is one of the several serious threats that severely hamper plant growth and productivity. Drought represents the shortage of water for a relatively longer period of time. However, depending on the type of plant species, generally, the continuous absence of water can kill a plant within a week. Soybean (*Glycine max* L.) is an important crop cultivated worldwide under a wide range of climatic conditions. However, water limitation directly affects the yield of soybeans by negatively affecting several physiological and morphological characteristics such as plant biomass and leaf pigmentation-related attributes resulting in significant economic loss [1, 2]. Excessive production of reactive oxygen species (ROS) and excessive lipid peroxidation as a result of prolonged drought conditions, lead to serious oxidative cell damage thereby disturbing the function of several key physiological pathways [3–5]. On the other hand, drought-resistant plants have the inherent ability to tolerate such conditions. For example, thick waxy layers, by rolling down the leaves to reduce the surface area for evapotranspiration, strictly regulating the movement of stomata and other natural openings to prevent water loss, reinforcement of the cells walls to prevent loss of water and electrolytes, and activation of antioxidative defense to scavenge ROS.

With the current change in global climate, hitherto soybean growing areas are now becoming unfit for its growth, for which drought is a key factor. A brief summary of the published literature on the effects of drought stress on soybean shows negative effects on the chlorophyll content, and relative water content with a significant increase in the accumulation of osmolytes, antioxidant potential, and membrane lipid peroxidation, reduction in pollen germination, changes in growth morphology and yield loss have been well studied [6, 7]. Drought-resistant soybean cultivars have been investigated to understand the mechanisms of tolerance and survival [2] which can be attributed to at least the partial recovery of photosynthetic traits [8, 9]. Apart from that, the effects of drought stress can be mitigated by various external applications.

Several studies have investigated the role of nitric oxide (NO) in mitigating different stressful conditions in plants [10–13]. Generally, NO is involved in maintaining different physiological as well as developmental responses in plants. In doing so, both endogenous and exogenous NO has been found to protect plants against the detrimental effect caused by stress-induced oxidative damage [14] by modulating gene expression and protein function. Nitric oxide regulates plant responses to drought, salinity and heavy metal stress [15–18]. However, NO is extremely unstable since it is a highly reactive molecule and has a very short half-life. In addition, exogenous NO treatment through NO donors is highly dose-dependent as it may lead to toxicity at a higher doses [18, 19]. For instance, the most studied NO donor, Sodium Nitroprusside (SNP) releases free cyanide in vivo both in plants and animals [20]. Hence, the S-nitrosothiol group derivative S-nitrosoglutathione (GSNO) is considered a potential NO donor. However, controlled and prolonged release of NO is considered a better alternative for protecting plants against various biotic and abiotic conditions [17]. When compared to chemical NO donors, nano-encapsulation is thought to be a promising and secure substitute [10, 12]. Generally. Published literature indicates the efficiency and effects of chitosan-based S-nitrosothiol nanoparticles under drought in sugarcane [21, 22], maize [10, 23], and *Heliocarpus* [24]. Therefore, our study focused on the chitosan-encapsulated GSNO nanoparticles (GSNONP) and their effect on the above and below-ground growth and development of soybean under drought stress with special emphasis on the antioxidant machinery and transcriptional regulation of key drought-response genes. Here, we have reported the synthesis and characterization of CS-GSNONPs. Chitosan is used for encapsulation since it is a biodegradable and biocompatible molecule [25, 26].

## Results

### Synthesis and characterization of GSNONP

**Fig. 1:**
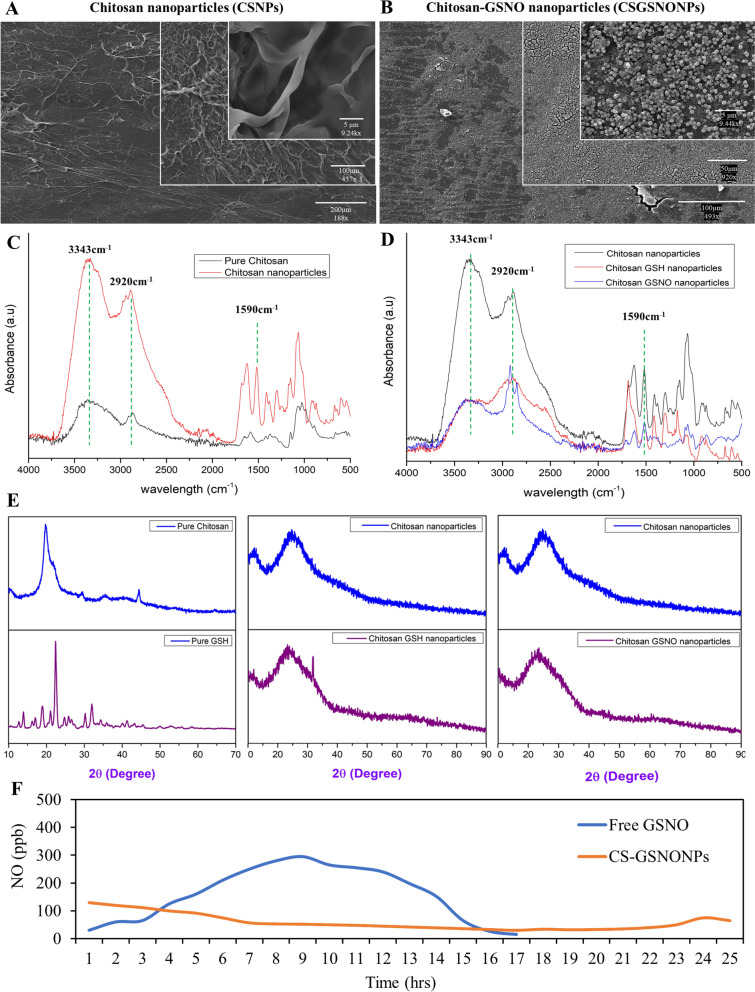
Characterization of chitosan nanoparticles and chitosan-GSNO nanoparticles. The CSNPs and CS-GSNONPs were characterized via scanning electron microscopy showing agglomerated morphology of CSNP (**A**) and spherical morphology of CS-GSNONPs (**B**). The nanoparticles were characterized using FTIR analysis detecting three unique peaks with wavenumbers of 3343 cm^−1^, 2920 cm^−1^, and 1590 cm^−1^ for pure chitosan and CSNPs (**C**). These peaks were also detected for CS-GSNONPs but with a significantly lower absorbance (**D**). Further characterization via XRD analysis also indicated unique characteristics for pure chitosan and chitosan-GSNO nanoparticles (**E**). Analysis of CS-GSNONPs for temporal kinetics of spontaneous gaseous NO release via Nitric Oxide analyzer (NOA280i, SIEVERS, USA) indicated a significantly slow and long-term release of NO as compared to free GSNO (**F**)

Chitosan nanoparticles have been under investigation for more than a decade now. Ionotropic gelation is one of the most widely used techniques for making chitosan nanoparticles [27] first described by P Calvo, C Remuñán-López, JL Vila-Jato and MJ Alonso [28] which is based on ionic cross-linking between inversely charged groups such as the positively charged chitosan and negatively charged polyanion groups of sodium tripolyphosphate (TPP). Here, we synthesized chitosan-GSNO nanoparticles (CS-GSNONPs) and later characterized them via scanning electron microscopy (SEM), fourier transformed infrared spectroscopy (FTIR), and X-ray diffraction (XRD) analysis (Fig. 1). Results of the dynamic light scattering (DLS) analysis indicated that the CS-GSNONPs had an average hydrodynamic size (HDS) of 383.3 nm, a polydispersity index (PDI) of 0.479 and zeta potential of 43 mV as compared to an average of 540.4 nm HDS, 0.587PDI and 49.30ζ for the chitosan nanoparticles alone (Supplementary Figure S1, and Table 1). The characterization of nanoparticles through scanning electron microscopy revealed the agglomerated morphology of CSNP (Fig. 1A) and spherical morphology of CS-GSNONPs (Fig. 1B). The agglomerated morphology of the CSNP can be attributed to higher dispersity index, whereas the spherical morphology after the saddition of the NO donor GSNO can be attributed to a reduction in the dispersity index.

**Table 1:** Dynamic Light Scattering (DLS) analysis of synthesized CSNPs and CS-GSNONPs

Nanoparticle	HDS (nm)	PDI or Đ	Zeta (ζ)-potential (mV)
CSNPs	540.4	0.587	49.3
CS-GSNONPs	383.8	0.479	43.0

Furthermore, FTIR analysis is commonly used to get information about the functional properties of nanochitosan that correlate with its structure and functional groups. The FTIR spectra of nanochitosan particles often have a peak at 3385 cm^−1^ indicating the symmetric and asymmetric stretching of the –NH_2_ and –OH groups wavenumbers [29]. We performed FTIR analysis for pure chitosan (CS) and chitosan nanoparticles (CSNPs) and observed three distinctive peaks with wavenumbers of 3343 cm^−1^, 2920 cm^−1^, and 1590 cm^−1^ for CSNPs (Fig. 1C). All three peaks were shared by the CSNPs, CS-GSHNPs, and CS-GSNONPs but with a significantly lower absorbance for the nano chitosan glutathione and S-nitrosoglutathione particles (Fig. 1D).

The diffraction pattern of pure samples, CS-NPs and CSNPs, GSH and NO-releasing nanoparticles GSHNPs and CS-GSNONPs were analyzed. XRD analysis of pure chitosan and pure GSH showed a characteristic peak at angles (2θ) 20 and 22.44 respectively [30, 31]. However, significantly broader peaks were recorded for nano-chitosan and nano-GSH particles (Fig. 1E). XRD analysis also indicated a successful combination of chitosan with GSH and GSNO. These results not only suggest good compatibility of chitosan nanoparticles with GSH and the NO donor GSNO but also indicate the amorphous nature of chitosan-GSNO nanoparticles which makes them useful for application in biological systems.

### Kinetics of NO release from free and nanochitosan-encapsulated GSNO

The kinetics of NO release from various NO-donors within biological systems play an important role in the absorption, longevity and bioactivity of nitric oxide. For this purpose, we determined the spontaneous release of gaseous NO from both free and nanochitosan-encapsulated GSNO (CS-GSNONPs) for up to 24 h. Results indicated that both NO donors experienced decomposition and released NO gas. The free GSNO released NO at a significantly higher rate, increasing almost exponentially after every hour reaching maximum values of 295 ppb within the first 8 h and then gradually decreased reaching 15 ppb after 16 h. CS-GSNONPs on the other hand, released significantly higher NO (around 130 ppb) for the first 2 h and gradually decreased to about 50 ppb holding steady for up to 24 h (Fig. 1F). This indicates the slow release of NO from the CS-GSNO nanoparticles for a significantly longer period of time compared to free GSNO which can have significant biological significance. These results also indicate that incorporation of GSNO in CS NPs can protect the NO-donor from rapid decomposition to ensure its optimal delivery, avoiding its negative impacts at higher dose.

### GSNONP mitigates drought stress *via* antioxidant defense

**Fig. 2:**
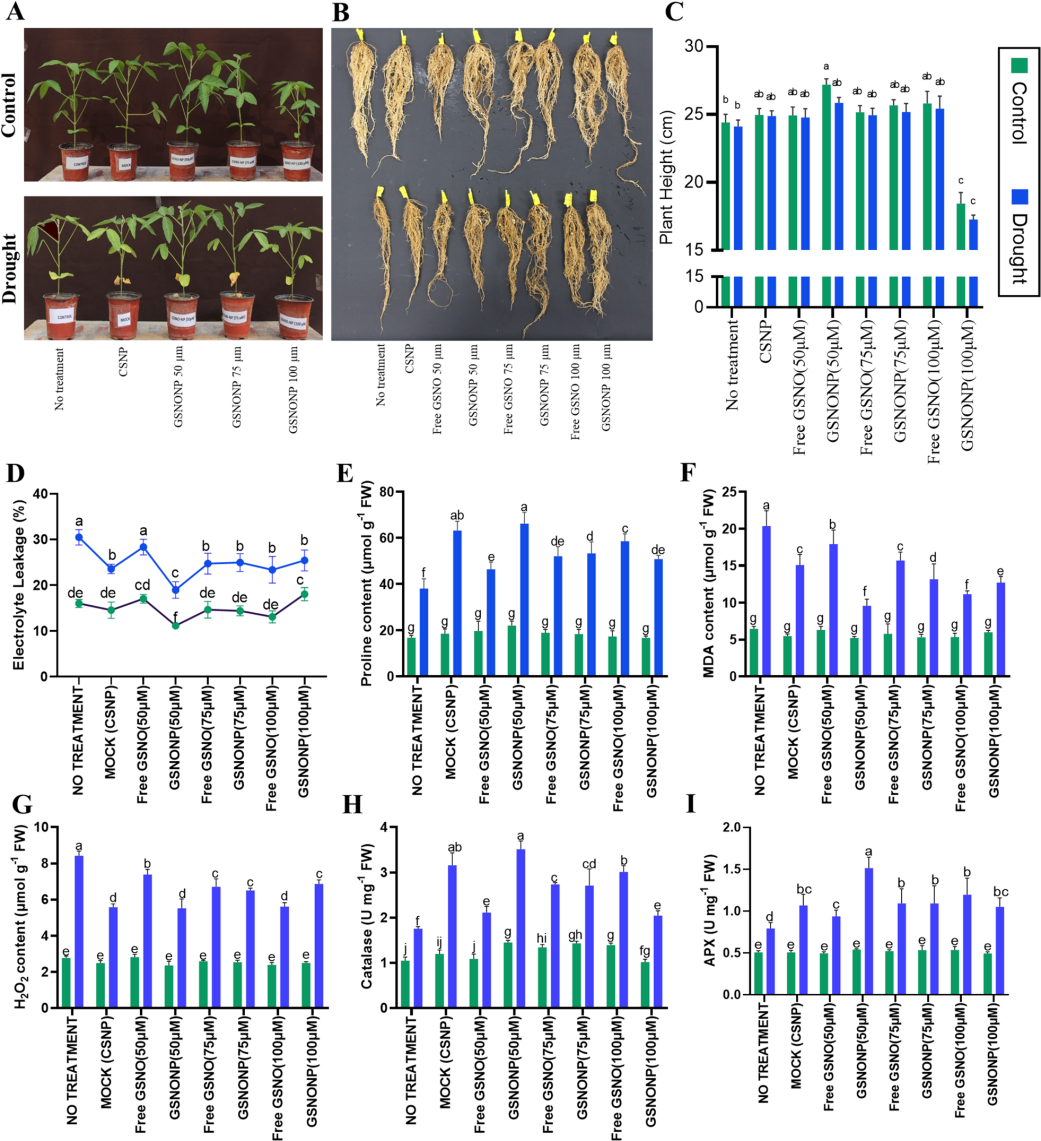
Chitosan-GSNO nanoparticles mitigate drought stress via antioxidant defense. CS-GSNONPs, had a significantly positive effect on soybean growth under irrigated as well as drought conditions (**A**) as reflected by an onverall increase in root biomass (**B**) and plant height (**C**) under both conditions. CS-GSNONPs provided protection by promoting cell integrity indicated by the reduced electrolyte leakage after treatment with 50μM GSNO (**D**) and via activation of antioxidant defense resulting in low proline content (**E**), MDA content (**F**), and H_2_O_2_ content (**G**) with a concomitant increase CAT activity (**H**), and APX activity (**I**). Each data point represents the average of atleast three replications. Error bars represent standard deviation. Means with significant difference were separated by DMRT following the analysis of variance (ANOVA) at *P* < .05

Application of CS-GSNONP had a significant effect on soybean growth under control as well as under drought stress. The 50 μM GSNO nanoparticles showed the optimum effect on plant phenotype above ground (Fig. 2A) whereas, all concentrations of GSNONOPs had a significantly positive effect on plant roots subjected to water stress (Fig. 2B). Drought stress resulted in stunting as well as yellowing of lower leaves whereas, plants treated with 50 μM GSNONP remained greened and were significantly taller than the normal plants as reflected by 11.5% and 7.3% more plant height under control and drought conditions (Fig. 2C). Interestingly, however, the higher concentration of GSNONP (100 μM) had negative effects on plant growth as it significantly reduced plant height by 24.5% and 28.5% under control and drought stress conditions, respectively (Fig. 2C). Contrastingly, the higher doses of 75 μM and 100 μM free GSNO as well as CS-GSNONP had a positive effect on the root growth (Fig. 2B). Similar results were obtained in another independent trial conducted in growth room conditions (Supplementary Figures S2 and S3).

To determine the effects of drought stress on soybean plants following the application of chitosan encapsulated GSNO particles, we measured electrolyte leakage (EL), MDA, H_2_O_2_ and lipid peroxidation in soybean leaves. Overall, the results indicated an average of 90% higher EL from the leaf tissues of plants subjected to drought stress than in the control plants, although the 50 μM CS-GSNONP concentration restricted the EL up to 37% (Fig. 2D). EL from plants treated with the 75 μM, and 100 μM GSNO and CS-GSNONP was statistically similar (Fig. 2D). Under normal irrigated conditions, the 50 μM CS-GSNONP application the electrolyte leakage was significantly lower than non-treated plants under the same conditions indicating the positive effects of chitosan GSNO nanoparticles on cell wall integrity at the cellular level. Likewise, the lowest proline and MDA contents were recorded in drought-stressed plants after application of 50 μM CS-GSNONPs (Fig. 2E and F) though these effects were not statistically significant in the irrigated plants. Furthermore, 50 μM CS-GSNONP and 75 μM CS-GSNONP resulted in a significantly low H_2_O_2_ content in the drought-stressed plants (Fig. 2G) with a concomitant increase of more than 90% in the catalase and ascorbate peroxidase (APX) activities in these plants (Fig. 2H and I).

### CS-GSNONPs promote the accumulation of photosynthetic pigments

Nano GSNO particles had a significantly positive effect on the accumulation of leaf photosynthetic pigments chlorophyll a, chlorophyll b, and total chlorophyll as well as the carotenoid content (Table 2). Soybean plants grown under normal irrigation conditions without any treatment had the lowest content of all the pigments. The highest *chlorophyll a* content (3.43 mg/g FW) was recorded in leaves of plants treated with 50 μM CS-GSNONP under normal conditions indicating that the application of GSNO nanoparticles can enhance the accumulation of chlorophyll and other pigments further contributing to plant development (Table 2). Similarly, we recorded the highest content of chlorophyll b in leaves of drought-stressed plants treated with CSNP (0.7084 mg/g FW) and with 100 μM free GSNO (0.7789 mg/g FW) (Table 2). However, the highest total chlorophyll content (3.8257 mg/g FW) and carotenoid content (0.1998 μg/g FW) was recorded in leaves of plants treated with 50 μM CS-GSNONP, under irrigated conditions (Table 2). The same pattern was observed in plants subjected to drought stress treatment where the CS-GSNONP treatment resulted in a statistically significant increase in the chlorophyll and carotenoid content (Table 2). Maximum chlorophyll a (2.89 mg/g FW), chlorophyll b (0.77 mg/g FW) were recorded in plants treated with 50 μM CS-GSNONP and 100 μM free GSNO, respectively whereas, the highest carotenoid content (0.18 mg/g FW) was recorded after treatment with the 50 μM CS-GSNONP (Table 2). The same treatment resulted in the highest total chlorophyll content (3.03 mg/g FW) in soybean plants subjected to drought stress (Table 2).

**Table 2:** Effect of CS-GSNONPs on leaf pigments in soybean subjected to drought

		***Chl a* (mg/g FW)**	***Chl b* (mg/g FW)**	**Total *Chl***	***Car* (mg/g FW)**
**Control**	**No Treatment**	2.36 ± 0.21^ef^	0.46 ± 0.03^de^	2.82 ± 0.18^efg^	0.13 ± 0.006^g^
	**Mock (CSNP)**	2.76 ± 0.17^cd^	0.70 ± 0.09^a^	3.47 ± 0.27^b^	0.17 ± 0.008^cd^
	**Free GSNO (50 μM)**	2.37 ± 0.18^ef^	0.41 ± 0.01^de^	2.79 ± 0.17^fg^	0.14 ± 0.02^efg^
	**CS-GSNONP (50 μM)**	3.43 ± 0.04^a^	0.38 ± 0.04^de^	3.82 ± 0.09^a^	0.19 ± 0.007^a^
	**Free GSNO (75 μM)**	2.57 ± 0.12^e^	0.61 ± 0.07^bc^	3.18 ± 0.21^c^	0.19 ± 0.01^ab^
	**CS-GSNONP (75 μM)**	3.01 ± 0.26^bc^	0.66 ± 0.02^a^	3.67 ± 0.23^b^	0.17 ± 0.02^bcd^
	**Free GSNO (100 μM)**	3.15 ± 0.52^b^	0.61 ± 0.07^abc^	3.77 ± 0.44^a^	0.19 ± 0.01^ab^
	**CS-GSNONP (100 μM)**	2.65 ± 0.06^de^	0.38 ± 0.02^e^	3.03 ± 0.08^cde^	0.15 ± 0.01^ef^
**Drought**	**Drought**	1.89 ± 0.24^g^	0.69 ± 0.01^a^	2.58 ± 0.23^g^	0.11 ± 0.009^h^
	**MOCK (CSNP)**	2.63 ± 0.14^de^	0.30 ± 0.00^f^	2.94 ± 0.14^def^	0.15 ± 0.01^de^
	**Free GSNO (50 μM)**	2.29 ± 0.16^ef^	0.40 ± 0.01^de^	2.69 ± 0.18^g^	0.13 ± 0.01^fg^
	**CS-GSNONP (50 μM)**	2.89 ± 0.23^bcd^	0.14 ± 0.00^g^	3.03 ± 0.22^cde^	0.18 ± 0.01^bc^
	**Free GSNO (75 μM)**	2.23 ± 0.15^f^	0.65 ± 0.03^ab^	2.93 ± 0.19^def^	0.12 ± 0.01^h^
	**CS-GSNONP (75 μM)**	2.80 ± 0.20^cd^	0.19 ± 0.003^g^	3.08 ± 0.19^cd^	0.16 ± 0.01^d^
	**Free GSNO (100 μM)**	2.05 ± 0.05^g^	0.77 ± 0.10^a^	2.87 ± 0.15^def^	0.13 ± 0.001^g^
	**CS-GSNONP (100 μM)**	2.31 ± 0.32^ef^	0.49 ± 0.06^cd^	2.82 ± 0.26^cdf^	0.13 ± 0.01^g^

Each data point represents the mean ± Data represented mean ± standard error of atleast three replicates. Means with significant difference were separated by DMRT following the analysis of variance (ANOVA) at *P* < .05. Different letters (a-h) represent significant differences among the means.

### Chitosan GSNO nanoparticles enhance root development under drought stress

**Fig. 3:**
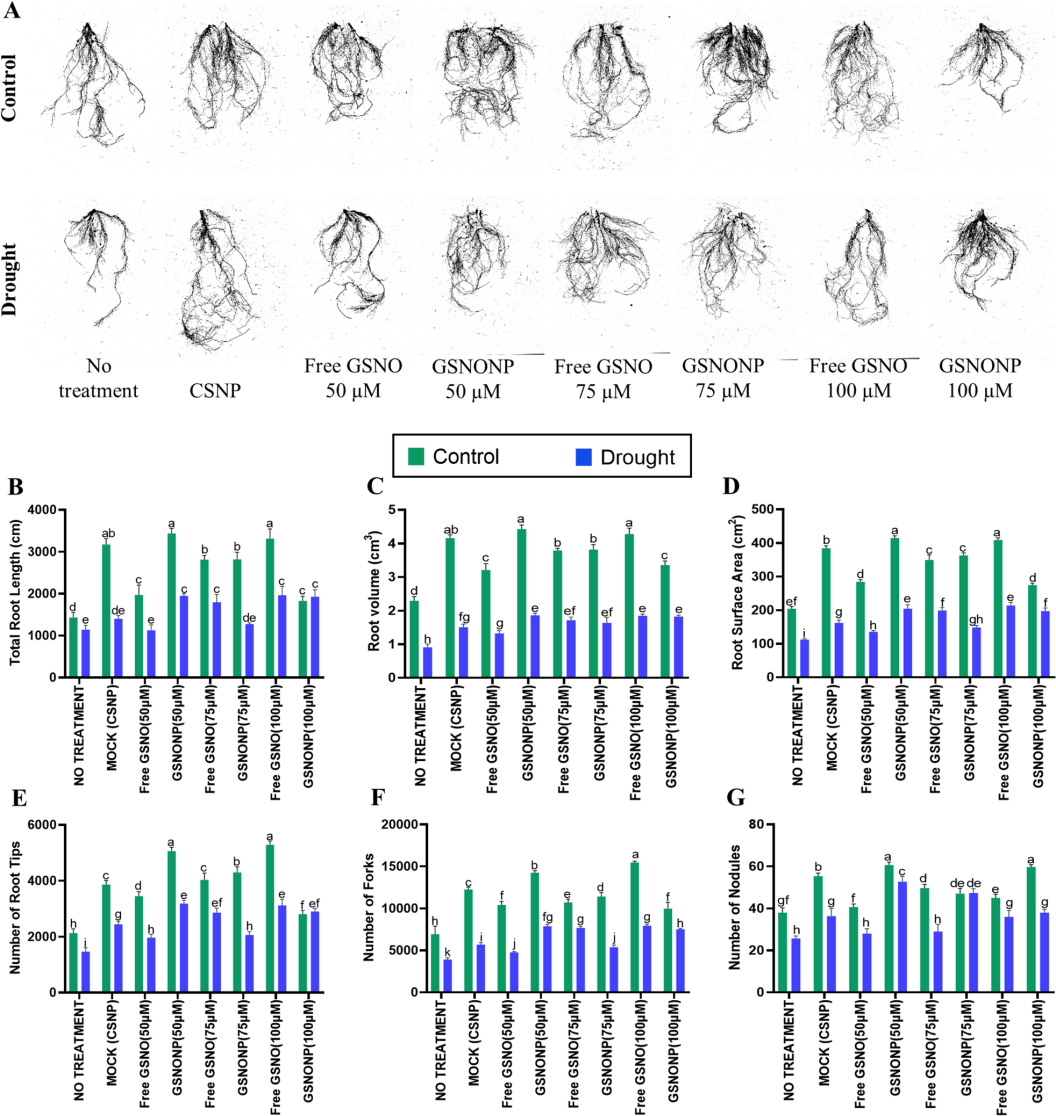
Effects of Chitosan-GSNO nanoparticles on soybean roots under drought stress. The various formulations of GSNO significantly enhanced root development-related traits under normal as well as drought stress conditions (**A**). A significant increase in root length (**B**) and volume (**C**) was recorded after the application of CS-GSNONPs. The 50 μM concentration of CS-GSNONPs and 100 μM free GSNO markedly increased the root surface area (**D**). The water-stressed soybean plants showed an increase of more than 100% in the number of root tips and forks (**E** and **F**) after application of CS-GSNONPs with a parallel increase in the number of nodules compared to the non-treated plants (**G**). Each data point represents the average of atleast three replications. Error bars represent standard deviation. Means with significant difference were separated by DMRT following the analysis of variance (ANOVA) at *P* < .05

Application of the different GSNO formulations significantly enhanced root development-related traits under normal as well as drought stress conditions (Fig. 3A). Among the various formulations, the 50 μM CS-GSNONPs and 100 μM free GSNO significantly increased the root length and volume by 93.2% and 86.8%, respectively (Fig. 3B and C) with more than 100% increase in the root surface area (Fig. 3D). However, under drought stress conditions, 100 μM CS-GSNONPs increased the root length and volume by 70.3% and 105.5%, respectively whereas, an increase of 71.8% and 103.8% in root length and volume, respectively was recorded in plants treated with 100 μM free GSNO as compared to the un-treated plants under the same conditions of drought (Fig. 3C). Likewise, the both formulations markedly increased root surface area by 82.3% and 90.6% under normal conditions (Fig. 3D). Furthermore, the water-stressed soybean plants, treated with 50 μM CS-GSNONPs and 100 μM free GSNO had more than 100% root tips and forks indicated a significant increase in root proliferation to explore more area in search of water (Fig. 3E and F). Interestingly, treatment of soybean plants with CS-GSNONPs increased the number of nodules compared to the non-treated plants, especially under the normal irrigated conditions (Fig. 3G). More specifically, the 50 μM and 100 μM CS-GSNONPs increased the nodule numbers by 59.6% and 57%, respectively in irrigated plants. Furthermore, under drought conditions, the number of nodules were significantly reduced in non-treated plants whereas, the CS-GSNONPs suppressed the negative impact of drought stress on nodule numbers resulting in the production of 105.2% and 84.4% higher number of nodules after treatment with 50 μM and 75 μM CS-GSNONPs, respectively (Fig. 3G). Similar results were obtained in another independent trial conducted in growth room conditions (Supplementary Figure S3).

### CS-GSNONPs ameliorate drought stress by regulating gene expression

**Fig. 4:**
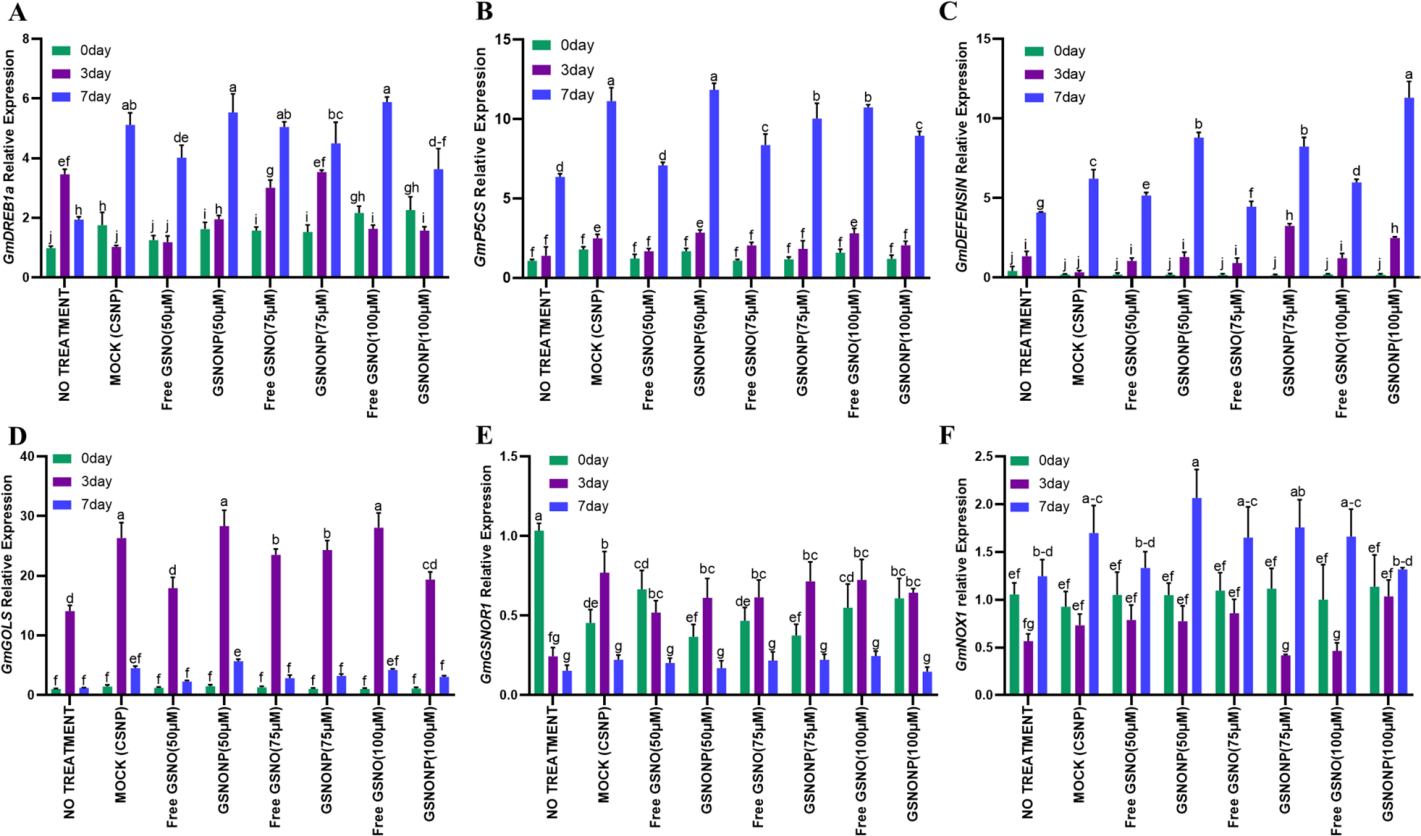
CS-GSNONPs ameliorate drought stress by regulating gene expression. Chitosan-GSNONPs resulted in statistically highest expression of the drought stress marker gene *GmDREB1a* 7 days of drought stress (**A**). Furthermore, the expression of the *GmP5CS* involved in proline biosynthesis and accumulation was also increased after application of GSNO nanoparticles (**B**). Similarly, expression of the soybean drought-induced proteinase inhibitor *GmDEFENSIN* markedly increased due to drought stress (**C**). Transcript accumulation of the soybean galactinol synthase *GmGOLS* reached maximum levels after 3 days and gradually dropped after 7 days of drought stress (**D**). Drought stress reduced the expression of the NO-related alcohol dehydrogenase *GmGSNOR1*, but the expression increased with the applicatin of chitosan and chitosan-encapsulated GSNO nanoparticles (**E**). On the other hand, the expression of *GmNOX1* increased after 7 days of the treatment with CS-GSNONPs compared to the non-treated control plants (**F**). Each data point represents the average of atleast three replications. Error bars represent standard deviation. Means with significant difference were separated by DMRT following the analysis of variance (ANOVA) at *P* < .05

We further explored the impact of chitosan encapsulated GSNO nanoparticles on the expression of drought responsive and ROS/RNS associated genes (Fig. 4). The 50 μM CS-GSNONPs and 100 μM free GSNO resulted in statistically highest expression of the dehydration responsive element *GmDREB1a* expression after 7 days of drought stress followed by 75uM CS-GSNONPs, and chitosan nanoparticles alone (Fig. 4A). Interestingly, the relative expression of *GmDREB1a* decreased on the 7^th^ day in plants subjected to drought stress only as the plants were completely wilted and almost dead by this time (Fig. 4A). However, all the other treatments significantly increased its expression under the same conditions. About 3 folds higher relative expression was recorded in plants treated with 50 μM CS-GSNONPs and 100 μM free GSNO after 7 days of drought stress compared to the plants subjected to drought stress only (Fig. 4A). This concludes that nitric oxide when delivered as 50 μM chitosan-encapsulated GSNO nanoparticles provides protection against drought-induced stress by enhancing the expression of soybean AP2 transcription factor family drought stress marker gene *DREB1a* (Fig. 4A).

Furthermore, we also investigated the relative expression of the *GmP5CS* that promotes proline biosynthesis and accumulation under abiotic stress conditions [32] in response to drought stress and application of GSNO nanoparticles. Results indicated the induction of *GmP5CS* by drought stress, especially at the late stages (7 days) (Fig. 4B). As expected, the 50 μM CS-GSNONPs induced the highest expression of this gene after 3 and 7 days of drought stress, followed by 75 and 100 μM CS-GSNONPs (Fig. 4B). Gene induction by free GSNO was similar to the GSNO nanoparticles at the earlier time points. These results suggest that GSNO nanoparticles provide protection against drought-induced oxidative damage by promoting the biosynthesis and accumulation of the osmolyte proline which can scavenge free radicals and is linked to enhanced enzymatic activity at the cellular level together with an increase in the expression of pro-metabolism genes and suppression of pro-catabolism genes [33].

The soybean drought-induced proteinase inhibitor *GmDEFENSIN* [(Genebank U12150) [34]] belongs to multi-member family of cysteine-rich proteins having strong antimicrobial activity and play key role in protection against abiotic stresses, especially drought stress [34–36] and is found in several plant species. Drought stress rapidly increased the expression of *GmDEFENSIN* with a more than a 50% increase in its expression after 7 days of drought stress (Fig. 4C). However, application of nanoparticles further increased its expression incrementally, with the highest expression recorded in plants treated with 100μM CS-GSNONPs after 7 days, followed by 75 and 50μM CS-GSNONPs at the same time point (Fig. 4C).

Similarly, the soybean galactinol synthase gene (*GmGOLS*) also responded to drought stress. However, its expression reached maximum levels after 3 days and gradually dropped after 7 days of drought stress indicating that *GmGOLS* is an early drought response gene as compared to *GmDREB1a*, *GmP5CS*, and *GmDEFENSIN* (Fig. 4D). Maximum relative expression was recorded in 3-days drought stressed plants treated with 50 μM CS-GSNONPs (4.6 folds increase in 3 days), followed by a 3.7-times increase by CSNPs and 3.4-times increase by 100 μM free GSNO in 3 days (Fig. 4D).

Furthermore, the expression of NO-related genes *GmGSNOR1* and *GmNOX1* was also investigated. Drought stress reduced the expression of the alcohol dehydrogenase *GmGSNOR1* (Fig. 4E). However, chitosan and chitosan-encapsulated GSNO nanoparticles significantly increased the expression of *GmGSNOR1* after 3 days of drought stress (Fig. 4E). On the other hand, in plants subjected to drought stress only, the expression of *GmNOX1* was reduced after 3 days of drought stress but increased after 7 days (Fig. 4F). The various concentrations of CS-GSNONPs increased the expression of *GmNOX1* in the same pattern where the expression was reduced after 3 days but increased significantly after 7 days (Fig. 4F).

## Discussion

Nanotechnology has been used in plant sciences to create, design, and engineer various nanoparticles (NPs) that can be used as antioxidants, antimicrobials, therapeutics, and diagnostics agents, and for the fabrication of nanosensors [37]. In the field of plant sciences, nanobiotechnology uses nanofibers, nanocapsules, and nanoparticles to improve plants through gene manipulation. These nanomaterials can act as gene carriers and or as substances that can trigger gene expression and control genetic material within plants. Applications of nanotechnology in agriculture include using nanoparticles to transfer genes or DNA into plants to develop insect-resistant varieties. It can also be used in food processing and storage to increase product shelf life and may even enhance the production of biomass-to-fuel [38]. Literature shows the use of different types of nanoparticles such as silver nanoparticles, titanium dioxide nanoparticles, zinc oxide nanoparticles, Cu-based NPs, iron oxide nanoparticles, carbon nanoparticles, and polymeric nanoparticles used as nano plant growth promoters, nano-fertilizers or -pesticides/herbicides, and as agrochemical encapsulated nanocarrier systems [39]. Metallic nanoparticles like gold and silver have also been widely used in plant sciences [39, 40]. Chitosan is a crustacean-acquired biopolymer emerging as a plant elicitor or bio-stimulant especially because it is non-toxic and biodegradable. Its application as nanoparticles (CSNPs) in agriculture for the enhancement of plant growth and development has been a recent topic of interest among plant scientists. Published literature indicates that CSNPs can provide protection against stresses via induction of the antioxidant defense, defense-related genes and production of secondary metabolites [41]. This tempted us to use nano chitosan for the delivery of NO through the ubiquitous nitric oxide donor GSNO. A Rosyada, WB Sunarharum and E Waziiroh [29] characterized CSNPs as edible coating material with a 585 nm particle size. In our study, the average particles size of CSNPs was 540.4 nm. However, the particle size of chitosan-GSNO nanoparticles was significantly smaller (383.8 nm Table 1). The interaction between GSNO and chitosan nanoparticles could change aggregation, crystalline structure and the overall topology of the nanoparticles thereby further decreasing the size of the combined particles. FTIR analysis is commonly used to characterize chitosan nanoparticles which are usually detected at FTIR spectra of 3385 cm^−1^ indicating the symmetric and asymmetric stretching of the –NH_2_ and –OH groups wavenumbers, for example by A Rosyada, WB Sunarharum and E Waziiroh [29] and at 3343 cm^−1^ in this study (Fig. 1). As can be seen from the FTIR-spectra, the CSNPs have a characteristic peak at 3343 cm^−1^ which was assigned to N-H extension vibration of primary amines, O–H stretching vibration, and H-bonding of the polysaccharide moieties of polymeric chitosan [42]. The characteristic peak at 2920 cm^−1^ [43] corresponds to symmetric aliphatic C-H vibration stretching, whereas the peak at 1590 cm^−1^ [44] spectrum corresponding to the carboxyl (C = O) group of primary amides and cross-linking of ammonium (-NH3) groups within tripolyphosphate (TPP). A similar IR spectrum for NO-releasing nanoparticles was observed for the prepared NO-releasing CS NPs with a minor shift in some absorption peaks, which might be due to an interaction between CS NP and GSNO while preparing NO-releasing nanoparticles. The role of NO in plant defense against a variety of biotic and abiotic stresses has been extensively studied in a variety of plant species under field conditions and in storage [45]. The various NO donors used by plant scientists include sodium nitroprusside (SNP), GSNO, and CySNO each with variable rates of absorption and kinetics of NO release. The CS-GSNONPs generated in this study released NO over a significantly longer period of time at lower rates which is much more desirable than normal NO donors (Fig. 1). This also highlights a greater utility of CS-GSNONPs for use as stable or long-term NO donors with a consistent rate of NO release. Our results demonstrated the key role of nitric oxide in drought stress mitigation in soybean. The growth and physiological attributes of soybean plants treated with GSNONPs under drought stress were enhanced to mitigate the deleterious effects of drought. Stress induced shift in growth patterns were suppressed by the nanoparticles. Especially the 50 μM concentration of CS-GSNONPs application resulted in a significantly healthier phenotype with maximum plant height, root biomass, pigments such as chlorophyll and carotenoids content (Table 2) and a more streamlined antioxidant defense system supported by concomitant change in the expression of key drought stress related genes. These findings also indicate a dose dependent response of soybean plants to chitosan encapsulated GSNO nanoparticles as the 50μM concentration was found to be statistically more suitable for most of the parameters than the 75 μM or 100 μM concentrations (Fig. 2). Similar findings were reported in soybean under heavy metal [25] and maize under salt stress [10] who used encapsulated S-nitroso-mercaptosuccinic acid.

Drought stress is known to cause significant oxidative damage at the cellular level resulting in higher electrolyte leakage from the cells together with membrane lipid peroxidation by various reactive oxygen species. Our results indicate significantly low electrolyte leakage in drought-stressed plants treated with 50 μM CS-GSNONPs. Furthermore, as described above, the osmolyte proline can scavenge ROS and is linked to enhanced enzymatic activity at the cellular level together with an increase in the expression of pro-metabolism genes and suppression of pro-catabolism genes [33] was increased by chitosan-GSNONPs indicating the production of osmoprotectants and improved cell integrity. M Rezayian, H Ebrahimzadeh and V Niknam [14] reported 60-80% increase in proline content following the application of NO whereas, an increase of 49% in the proline content was reported in *Salvia* [46] and maize [47] following the application of CSNPs. In addition, we also recorded a significant reduction in MDA and H_2_O_2_ contents following the application of CSGSNONPs to drought-stressed plants. In this case, 50 μM CS-GSNONPs provided a protective effect equivalent to 100 μM free GSNO. Higher level of antioxidative activity promotes detoxification of various reactive oxygen species. In a similar study, foliar spray of sugarcane plants with GSNONP under drought induced the antioxidative defense system [21]. In another study higher CAT and APX activity were recorded under drought stress following soil application of NO-chitosan nanoparticles [25]. Chitosan-GSNO nanoparticles enhanced several root related traits such as total length, volume, surface area, number of root tips and forks. Furthermore, CS-GSNONPs also increased the number of nodules which is a very important character for legume crops. This effect might be related to the prolonged and consistent release of NO by the CS-GSNONPs (Fig. 3). However, the impact of NO, NO donors or NO releasing nanoparticles on root architecture and other traits is largely unknown. HC Oliveira, BC Gomes, MT Pelegrino and AB Seabra [10] reported significnatly better root growth of maize plants after application of NO-based chitosan nanoparticles and NM Silveira, AB Seabra, FC Marcos, MT Pelegrino, EC Machado and RV Ribeiro [23] recorded maximum root-shoot ratio in sugarcane after application of GSNONP under drought stress conditions.

Plant response to abiotic stresses involve the activation of different transcriptional, translational and other biochemical machinery. However, its efficiency and magnitude depend upon the resistance of the host and the intensity and duration of the stress. Prolonged, intense stress often result in permanent damage to the host plants. The chitosan GSNO nanoparticles used in this study provided protection against drought induced damage via activation of the related transcriptional machinery (Fig. 4). The AP2 transcription factor family gene DREB are well-known drought stress marker genes as they positively regulate plant tolerance to drought stress. Owing to the significantly enhanced phenotypic response of the CS-GSNONPs treated soybean plants to drought stress, the significant increase in the expression of *GmDREB1a* was expected. Furthermore, the higher accumulation of proline content was supported by an increase in the expression of *GmP5CS* which is involved in proline biosynthesis in plants. In soybean, nitric oxide supplementation resulted in increased proline synthesis due to enhanced *GmP5CS* expression [48]. On the other hand, chitosan supplementation enhanced proline content in rapeseed under abiotic stress condition [49]. With further increase in the expression of *GmDEFENSIN*, *GmGOLS*, CS-GSNONPs enhanced soybean drought tolerance via modulation of the transcriptional machinery. These genes are known to play a key role in the drought tolerance of soybean plants and are up-regulated by water shortage [36]. Similarly, [50] reported an increase in the expression of *GmNOX1* in soybean due to abiotic stress. Taken together, these results indicate the significant utility of chitosan-GSNO nanoparticles for growth promotion in plants under drought stress conditions.

## Conclusions

Though nanomaterials had been studied extensively in biomedical field, application in plant sciences has remained underexplored. Our investigation stated that chitosan encapsulated GSNONP not only increased the physiomorphological, biochemical, root morphological characteristics remarkably but also positively regulated gene related transcripts in response to drought. In short, such approach might have optimistic implication to construct tolerance in soybean plants subjected to abiotic stress. Besides, we could conclude, both encapsulated GSNO and free GSNO alleviated the deleterious effect caused by water deficit. However, GSNONP 50μM increased and improved tolerancy to drought since it ensured NO supplementation for prolonged time.

## Methods

### S-nitrosoglutathione (GSNO) synthesis

GSNO was synthesized by mixing equimolar concentrations (200mmol/L) of reduced glutathione (GSH) and sodium nitrite (NaNO_2_) in 0.5mol/L HCl. The resultant mixture was stirred in an ice bath for 45min in the dark and precipitated using acetone. The precipitates were filtered and washed thrice with 5ml each of ice-cold distilled water, acetone, and finally with 1ml ethoxyethane. The precipitates were freeze-dried overnight to make GSNO and stored in the dark [51].

### Synthesis and application of chitosan nanoparticles (CSNP) and GSNO-CSNP

CSNPs were prepared via the ionotropic gelation method as described [52–54]. Briefly, 1.7mM (CS; Mw ~ 190–370 kDa, deacetylation ≥ 70%) was dissolved in 1% acetic acid. Then, 1.3mmol/L GSH solution was added to the suspension and allowed and mixed on a magnetic stirrer for 90 min at room temperature. Next, 0.6mg/mL tripolyphosphate (TPP) solution was added drop-wise to CSGSH suspension, ensuring a volumetric ratio of 3CS-GSH:1TPP, and stirred for another 90 min. To get GSNO-CSNPs, NaNO_2_ (equimolar concentration to GSH) was added to the GSH-CSNP suspension. The resulting clear solution was allowed to homogenize and react for 90 min in the dark at room temperature and used directly for the tretments. For foliar treatments, the CSNP and CS-GSNONPs were diluted using deionized water to obtain the required concentrations.

### Dynamic Light Scattering (DLS) measurements

The mean hydrodynamic size, polydispersity index (PDI), and zeta potential of CSNPs and GSNO-CSNPs were measured using dynamic light scattering (DLS) [55], using Malvern nano-zeta sizer (Malvern Instruments Co. UK). Measurements were done at 25°C at 173° angle in DTS1070 capillary cells with a path length of 10mm with water as dispersant at an equilibrium time of 120 s.

### Scanning Electron Microscopy (SEM) observation

The morphology of NPs was investigated using a scanning electron microscope (SEM) [56]. The samples were mounted on aluminum stubs, dried using critical point drying (CPD, Emitech), and then coated with gold using a sputter coater model E-1010 (Emitech) and observed via scanning electron microscope, (S-2700, Hitachi Ltd, Tokyo, Japan).

### Fourier Transform Infrared (FTIR) analysis

CSNPs, CS-GSHNPs, and CS-GSNONPs were subjected to FTIR analysis [57]. Aqueous suspensions were freeze-dried for 24 h before FTIR examination to get powdered nanoparticles. CSNPs, GSH-CSNPs, and GSNO-CSNPs were titrated with pure potassium bromide (KBr) and processed into translucent pellets via mechanical pressing and examined in a Bomen B-100 spectrometer. FTIR spectra were obtained with a resolution of 4 cm^−1^ and ranged from 400 to 4000 cm^−1^.

### XRD analysis

For the XRD analysis, 200 mg were freeze dried and finely powdered samples were deposited on to a glass substrate of 2cm^2^. XRD patterns were recorded using Cu Kα radiation (λ = 1.5406 Å) with nickel monochromator in the 2θ range from 5.08° to 89.98° using Panalytical Empyrean X-Ray diffractometer (Malvern Panalytical Ltd. Malvern, United Kingdom).

### Kinetics of NO gas released from GSNO and CS-GSNONPs

Nitric oxide gas emission from free GSNO and CS-GSNONPs were measured using the Nitric Oxide Analyzer (NOA280i, SIEVERS, USA) for 24 h continuously at 25 °C [23, 26].

### Plant material, growth conditions, and treatments

The seeds of soybean cultivar Pungsannamul were obtained from the Plant Genetics and Breeding laboratory of the college of Agriculture and Life Sciences, Kyungpook National University, South Korea. The seeds were surface sterilized in 12.5% sodium hypochlorite (NaHCl) and washed with sterilized distilled water. Two seeds each were sown in pots containing sandy soil in a greenhouse 14/10 h of day/night cycle and a temperature of 28 °C ± 2 °C arranged in a completely randomized design with three replications. The plants were treated with water (control), CSNPs (mock), and 50 μM, 75 μM, and 100 μM each of GSNO, and CS-GSNONPs applied as 5 mL solution through foliar spray and via soil drenching for 10 consecutive days. Data on different phenotypic characters was recorded and the plants were subjected to drought stress by withholding water for seven consecutive days, whereas the control plants were irrigated normally. Phenotypic responses of plants to drought stress were recorded and samples were collected for further analysis. After seven days of drought stress, plants were re-watered for recovery. Plants were uprooted at the V6 stage to record data on root-related traits.

### Plant height and chlorophyll content

Plant height was measured using a measuring scale by measuring the height of the plants from the crown to the tip of the plants. Chlorophyll a, b and carotene contents were determined as described by J Hiscox and G Israelstam [58–60]. Briefly, for chlorophyll extraction, 100 mg of fresh leaf samples were immersed in 20 ml of Dimethyl Sulfoxide (DMSO), incubated for 4 h at 65 °C and allowed to cool at room temperature. Absorbance was recorded at 663, 645, and 470 nm in a UV spectrophotometer (UV-1280, Shimadzu Japan). Chlorophyll a, b, and carotene contents were calculated based on Arnon’s equations given;

$${Chla}_{(mg/g\;fresh\;weight)}=\left[\left(12.7\;\times\;A_{663}\right)-\left(2.69\;\times\;A_{645}\right)\right]\times\frac{Vol}{W\;\times\;1000}$$

$${Chlb}_{(mg/g\;fresh\;weight)}=\left[\left(22.9\;\times\;A_{645}\right)-\left(4.68\;\times\;A_{633}\right)\right]\times\frac{Vol}{W\;\times\;1000}$$

$${Total\;Chlorophyll}_{(mg/g\;fresh\;weight)}=(20.08\;\times\;A_{645\;}+\;8.02\;\times\;A_{663})\;\times\frac{Vol}{W\;\times\;1000}$$

$${Car}_{\left(mg/g\;fresh\;weight\right)}=\frac{1000A_{470}\;-\;1.90{Chl}_{a\;}-\;63.14{Chl}_b}{214}\;\times\;\frac{Vol}{W\;\times\;1000}$$

Where;

V = volume of extract (ml)

W = fresh weight of the samples (g)

Car = Carotenoids

### Measurement of electrolyte leakage, ROS, lipid peroxidation, and antioxidant activity

Electrolyte leakage was measured by cutting 2 leaf discs of 1 cm diameter from control and treated plants. The two leaf discuss were cut from at least three leaves of every plant. The leaf discs were incubated in 5 mL of deionized water for 2 h. Electrolyte leakage (EL1) was measured using a portable conductivity meter (HURIBA Twin Cond B- 173, Japan). The leaf discs were then autoclaved at 120 °C for 20 min and electrolyte leakage (EL2) was measured again. Net electrolyte leakage was calculated using the following formula;

$$EL\;\left(\%\right)=\frac{EL1}{EL2}\;\times\;100$$

Proline and H_2_O_2_ contents were measured as described by L Bates, Ra Waldren and I Teare [61] using a standard curve. For this purpose, 0.5 g finely macerated leaf tissues from the control and treated plants were homogenized in 10 ml 3% sulfosalicylic acid and centrifuged for 10 min at 15000 rpm. Two ml of the supernatant was mixed with 2 ml each of acid ninhydrin and glacial acetic acid in fresh tubes and incubated at 100 °C for 1 h. The reaction was terminated on ice. The reaction was extracted with 4 ml toluene, and mixed vigorously for 20 s. The chromophore containing toluene was aspirated from the aqueous phase and warmed to room temperature. The absorbance was recorded at 520 nm using only toluene as a blank. Proline content (μmol/gFW) was determined from a standard curve using the following formula;

$$\mathrm{Proline}\;{\mathrm{content}}_{(\mathrm\mu\mathrm m\mathrm o\mathrm l/\mathrm{gFW})}=\frac{\lbrack(\mu g\;proline/ml\;\times\;ml\;toluene)/{115.5\mu g\mu mole}^{-1}\rbrack}{weight\;of\;sample(g)/5}$$

H_2_O_2_ contents were measured spectrophotometrically after a reaction with potassium iodide (KI) as described by V Alexieva, I Sergiev, S Mapelli and E Karanov [62]. Leaves were homogenized in 0.1% trichloroacetic acid (TCA) and centrifuged for 10 min at 15000 rpm. Next, 500μl of the supernatant was mixed with an equal quantity of 100 mM potassium phosphate buffer and 2 ml of 1 M KI reagent for 1 h in the dark. A blank reaction was also set up using 0.1% TCA without any leaf samples. Absorbance was recorded at 390 nm and the amount of H_2_O_2_ was calculated using a standard curve prepared with known concentrations of H_2_O_2_.

Malondialdehyde (MDA) measurement has long been used as a maker of lipid peroxidation in studies related to oxidative stress and redox signaling in plants, especially in response to abiotic and biotic stresses. We measured MDA content in the control and treated plant samples as described by RL Heath and L Packer [63]. Briefly, leaf samples were homogenized in 5% Thiobuteric acid (TBA) and centrifuged for 10 min at 15000 rpm. The supernatants were mixed in 20% trichloroacetic acid (TCA) for 25 min at 95 °C. Absorbance was first recorded at 532 nm and then at 600 nm. The A532 values were corrected by subtracting the absorbance at A600. MDA content was calculated by using an extinction coefficient of 155 mM^−1^ cm^−1^ and expressed as micromole of MDA g^−1^ FW.

Catalase activity was measured in a 1 ml reaction mixture containing of 100 mM sodium phosphate (Na_2_HPO_4_) buffer (pH 7.4), 10 mM H_2_O_2_ and 20 μg of protein extract from the control and treated plant samples. Catalase activity was detected at 240 nm with an extinction coefficient of 39.4 mM [64]. Similarly, APX activity was detected in a reaction mixture containing 50 mM phosphate buffer, 0.1 μM EDTA, 0.5 M ascorbate and 1 mM H_2_O_2_ at 290 nm wavelength with an extinction coefficient of 2.8 mM [65].

### Root sampling and analysis

Plants were uprooted, the soil was removed carefully and the roots were gently washed and dried using paper towels. The plant roots were evenly spread and scanned using the EXP12000XL scanner (Epson, Japan). Images were analyzed for root morphology using the WinRHIZO™ Pro analysis software (Regent Instruments Inc. Canada).

### Quantitative real-time PCR analysis

The quantitative real time PCR was performed for analyzing of relative gene expression of *GmDREB1a*, *GmP5CS, GmDEFENSIN, GmGOLS, GmGSNOR1* and *GmNOX1* using the primers listed in Supplementary Table S1. Leaf samples were ground in liquid nitrogen and RNA was extracted using the Trizol reagent (Invitrogen, USA) Next, 1μg of RNA was used to prepare cDNA using the BioFact™ RT-Kit (BioFact, Korea) following manufacturer’s instructions. Two-step quantitative real-time PCR was performed in EcoTM real-time PCR machine (Illumina, San Diego, CA, USA) using the 2X Real-Time PCR Master Mix including SYBR®Green l (BioFact, Korea) with an initial and subsequent cycle denaturation steps at 95 °C for 15 min and 20 s, respectively and simultaneous primer annealing and extension at 55 °C for 40 s. The Elongation factor 1-beta 2 reference gene and no-template controls were used as comparative controls.

### Statistical analysis

All experiments were performed in replicates. Data were recorded, means and standard deviation were calculated in Microsoft Excel. The data were analyzed using the student’s t-test at *P* ≤ 0.05 in Microsoft Excel (MS Office Professional Plus 2019) and ANOVA wherever required. ANOVA was performed using GraphPad Prism version 9.0.0 (San Diago, CA, USA). Significantly different means were separated using the Duncan’ts multiple range test (DMRT) in SAS version 9.4 (SAS Institute Inc., Cary, NC, USA).

### Supplementary Information

**Additional file 1: Supplementary Figure S1.** Characterization of chitosan nanoparticles (CSNPs) and chitosn-GSNO nanoparticles (CS-GSNONPs).

**Additional file 2: Supplementary Figure S2.** Chitosan-GSNO nanoparticles mitigate the effects of drought stress on the above and below-ground parts of soybean plants grown under growth room conditions.

**Additional file 3: Supplementary Figure S3.** Effects of Chitosan-GSNO nanoparticles on soybean subjected to drought stress under growth room conditions.

**Additional file 4:** **Table S1. **List of primers used for quantitative real-time PCR.

## Data Availability

All data is available within the manuscript. Any other information if required will be made available by the corresponding author on request.
