# Supplementary figures and images for: Chitosan-GSNO nanoparticles: a positive modulator of drought stress tolerance in soybean
Source: BMC Plant Biol. 2023 Dec 11;23:639. doi: 10.1186/s12870-023-04640-x (PMC10712192; doi:10.1186/s12870-023-04640-x)

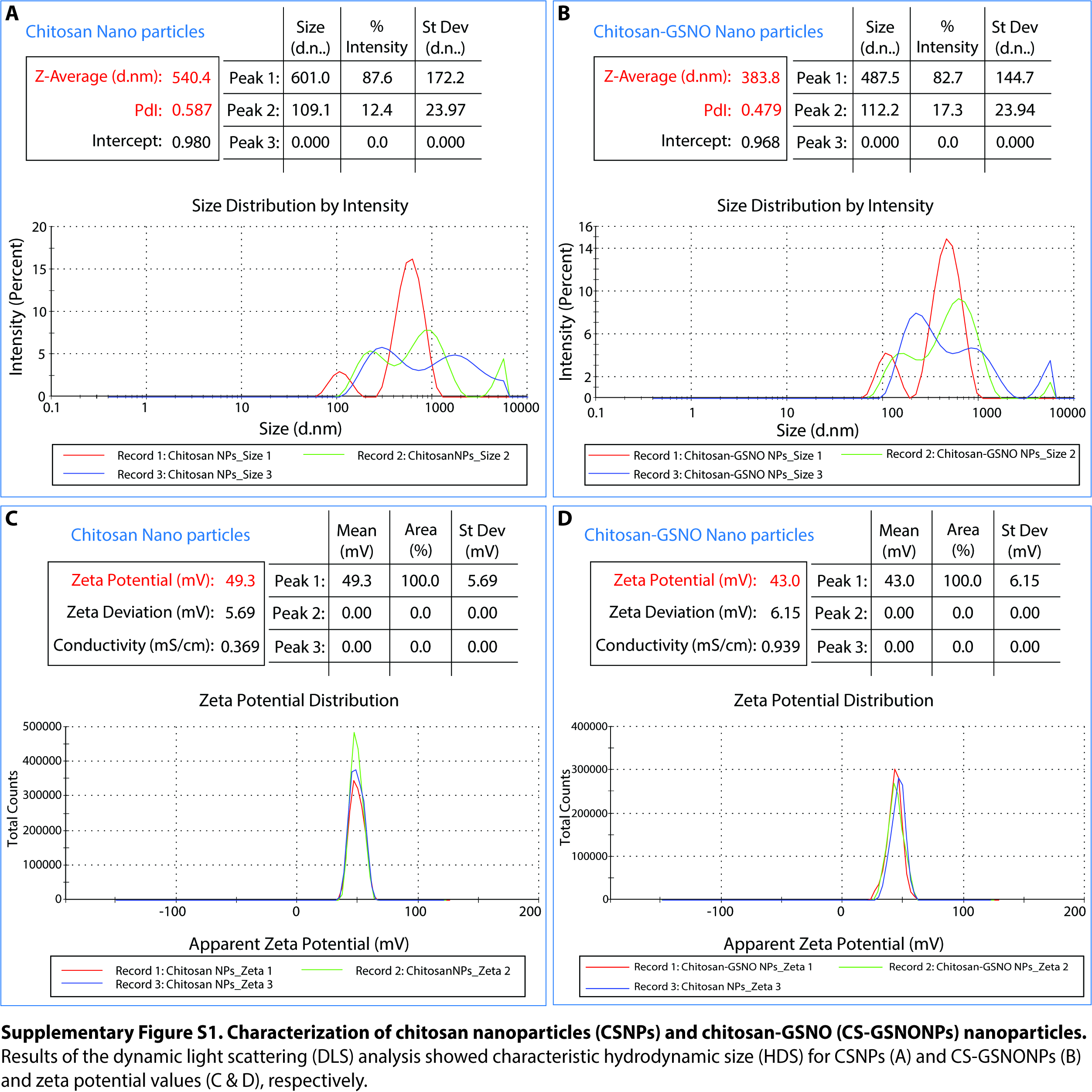

Supplement: Supplementary file 1 — Additional file 1: Supplementary Figure S1. Characterization of chitosan nanoparticles (CSNPs) and chitosn-GSNO nanoparticles (CS-GSNONPs). [file 12870_2023_4640_MOESM1_ESM.tif]

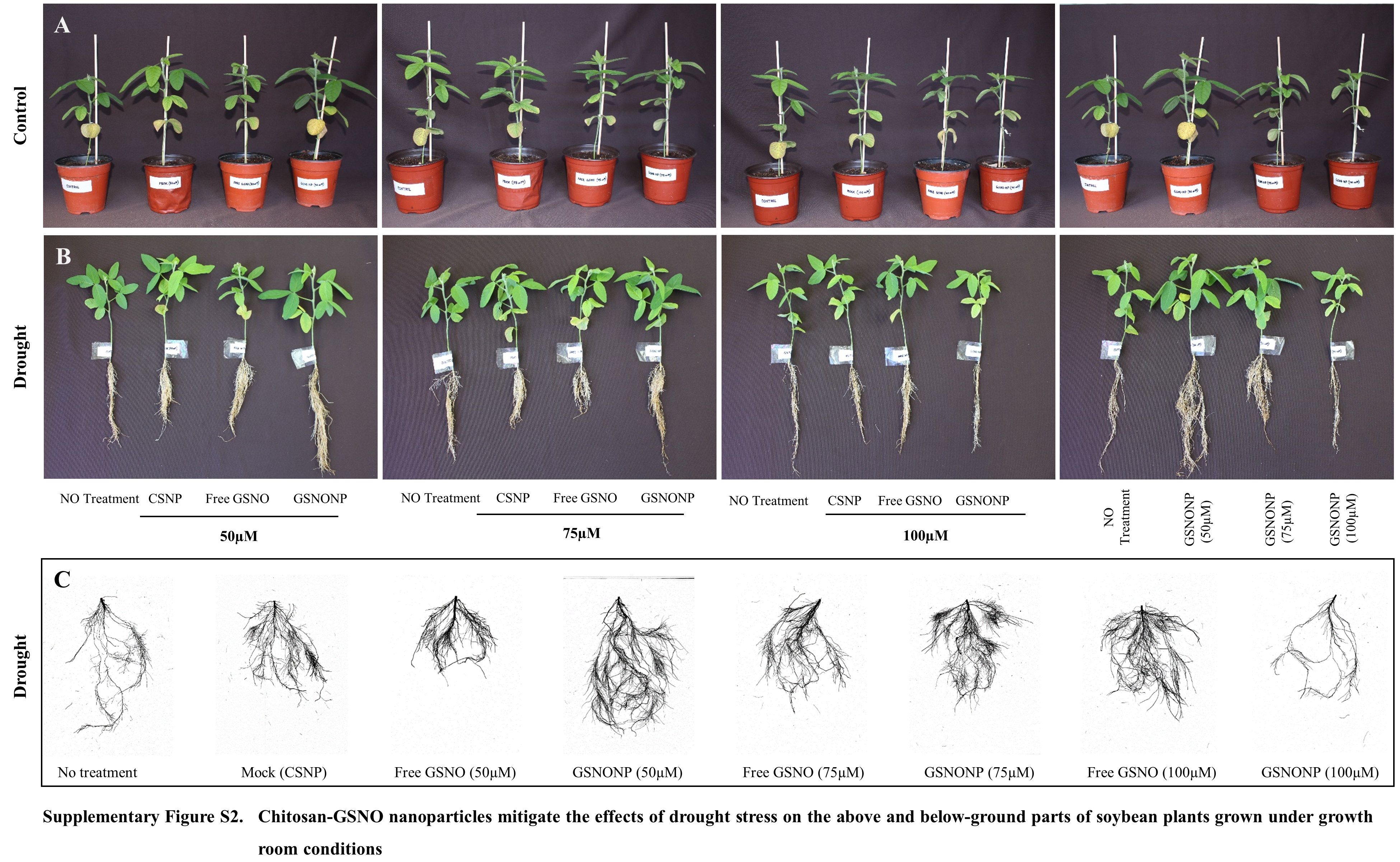

Supplement: Supplementary file 2 — Additional file 2: Supplementary Figure S2. Chitosan-GSNO nanoparticles mitigate the effects of drought stress on the above and below-ground parts of soybean plants grown under growth room conditions. [file 12870_2023_4640_MOESM2_ESM.tif]

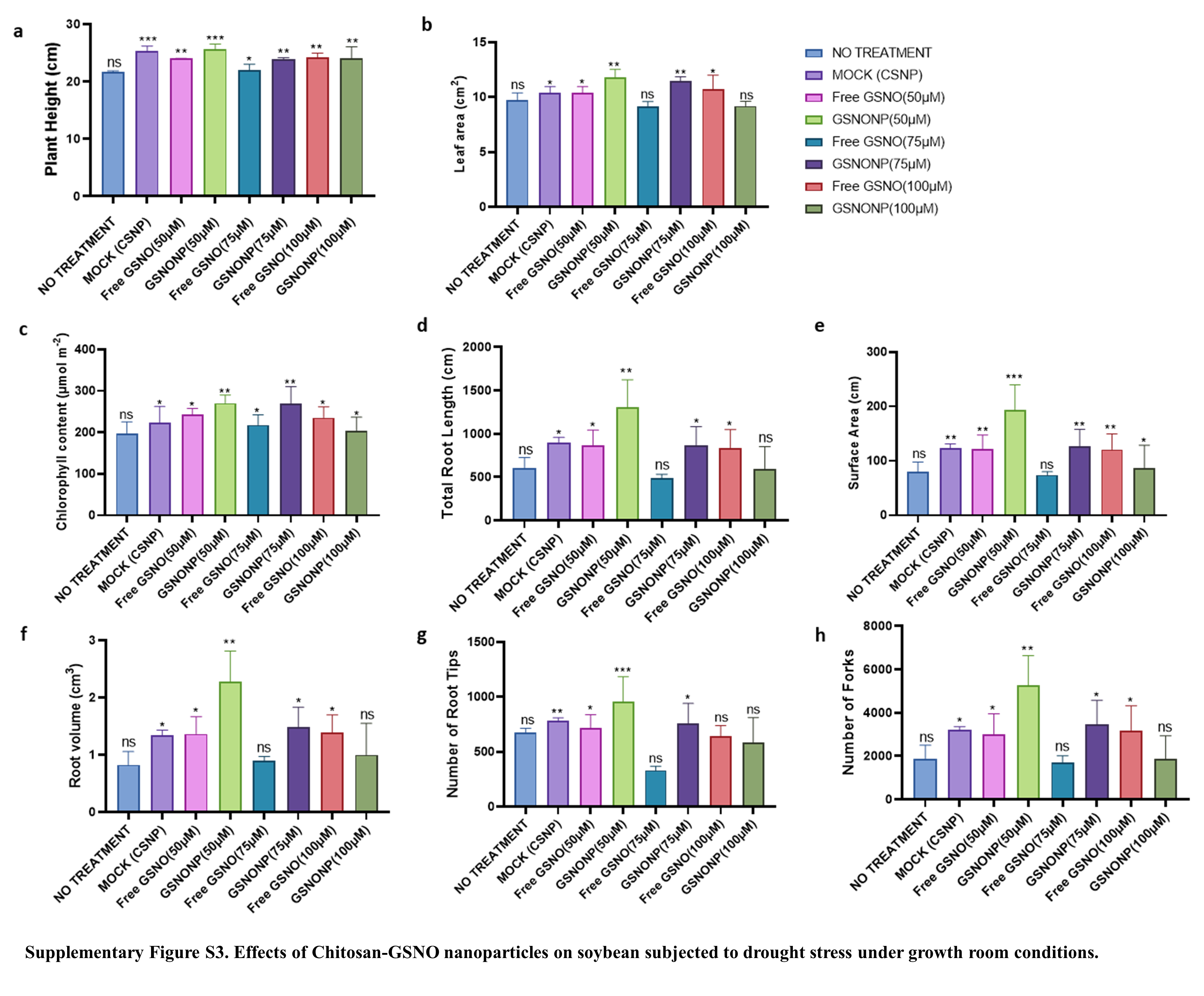

Supplement: Supplementary file 3 — Additional file 3: Supplementary Figure S3. Effects of Chitosan-GSNO nanoparticles on soybean subjected to drought stress under growth room conditions. [file 12870_2023_4640_MOESM3_ESM.tif]
