# Supplementary material for: Chitosan-GSNO nanoparticles: a positive modulator of drought stress tolerance in soybean
Source: BMC Plant Biol. 2023 Dec 11;23:639. doi: 10.1186/s12870-023-04640-x (PMC10712192; doi:10.1186/s12870-023-04640-x)
Supplement: Supplementary file 4 — Additional file 4: Table S1. List of primers used for quantitative real-time PCR. [file 12870_2023_4640_MOESM4_ESM.docx]

**Table S1. List of primers used for quantitative real-time PCR.**

|  | **Gene Name** | **Description** | **Sequence (5ʹ-3ʹ)** |
| --- | --- | --- | --- |
| 1. | *GmDREB1a* | dehydration-responsive binding protein | F: CGACCAGGAGGGCAGTGAT  R: GCTTTTCGGCGAATGGAAT |
| 2. | *GmDEFENSIN* | drought-induced proteinase inhibitor | F: TTTGAGTGACACCAACTGTGG  R: AACAATGTTTGGTGCAGAAGC |
| 3. | *GmP5CS* | D1 -pyrroline-5-carboxylase synthetase (involved in proline synthesis) | F: TGTCTCTCAGATCAAGAGTTCCAC  R: CAGCCTGCTGGATAGTCTATTTTT |
| 4. | *GmGolS* | Galactinol synthase | F: GTGACCACAACCCTTCCTCC  R: TCAACGTTCTCAGGGTGACG |
| 5. | *GmGSNOR1* | S-Nitrosoglutathione Reductase /alcohol dehydrogenase class-3 | F: TGTTGGGTTGCTTTTCCCCT  R: TCTTTCTGATCACAAGTTTGCCA |
| 6. | *GmNOX1* | NADPH Oxidase (NO overproducer) | F: CAGAGCGCGGCTTTCACTTT  R: TCTTTCTGATCACAAGTTTGCCA |
| 7. | *GmELF1b* | Reference gene (elongation factor 1-beta 2) | F: CCACTGCTGAAGAAGATGATGATG  R: AAGGACAGAAGACTTGCCACTC |
